# Overcoming barriers to effective feedback: a solution-focused faculty development approach

**DOI:** 10.5116/ijme.5f7c.3157

**Published:** 2020-10-23

**Authors:** Samar McCutcheon, Anne-Marie Duchemin

**Affiliations:** 1 Department of Psychiatry, The Ohio State University College of Medicine, Columbus, OH, USA

**Keywords:** Effective feedback, solution focused, medical education, USA

## Introduction

Data supporting the importance of feedback in medical education has increased since the 1980s^1^ and education research has established that feedback is crucial to improvements in trainee performance, professionalism, documentation, and communication skills.^2^^-^^4^ However, supervisors continue to experience barriers to delivering feedback to trainees. This inability to deliver effective feedback not only hinders the learning experience but may even negatively impact the mental health of trainees.^5^ We sought to review feedback research, identify the most common feedback barriers experienced, and generate evidence-based solutions for each barrier. This knowledge can inform individual faculty development and assist supervisors in gaining the skills they need to deliver feedback effectively.

### Overview of the approach

Our proposed approach to individualized faculty development for feedback begins with supervisors engaging in a self-inventory of feedback experiences. Supervisors should explore and identify which barriers they experience during feedback delivery. In a survey of 236 supervisors at our large academic medical center, the four most commonly cited barriers were: lack of time, fear of damaging rapport, trainee resistance and lack of comfort with feedback delivery. We believe faculty development must focus on these barriers and solutions to overcoming them in order to optimize the feedback experience for supervisors and trainees.

Of the feedback barriers, time constraints are most commonly cited.^6^ Research suggests implementing a variety of feedback methods during the learning experience can address this barrier. Existing evidence-based feedback methods include formal feedback sessions (individual sessions with the trainee), real-time feedback (provided during the course of clinical care) and written evaluations. Formal feedback sessions are challenging as they require the most time commitment from supervisors.^7^ Data supports using formal sessions at the beginning of the learning experience to set goals and expectations, and at the end of the learning experience to assess the progress achieved during the learning experience.^8^ Real-time feedback provided during clinical care requires minimal time and can remain high yield. To further optimize time, positive feedback can be delivered in a group-setting without negatively impacting the trainee.

Despite adequate time for feedback, supervisors may find that fear of damaging rapport with their trainee is a barrier to feedback delivery. Research has shown it is possible that providing feedback can damage the training relationship; the solution to this barrier is to create a safe environment for feedback delivery.^9^ Kraut and colleagues identify normalizing a culture of feedback as a key component of creating this safe environment.^10^ Feedback must be introduced early in the learning experience and acknowledged as an integral part of the training. Bing-You and colleagues propose when supervisors show an investment in the trainee's growth, they can provide feedback from a genuine place of caring.^11^ Supervisors can also be trustworthy mentors by modeling desired behaviors, consistent with survey results demonstrating trainees respect supervisors who "practice what they preach".^12^ When providing feedback, the research emphasizes the importance of focusing on the trainee's behavior rather than their personal characteristics. Instead of delivering feedback as an objective statement, feedback theory supports sharing how the trainee's behavior made supervisors feel.^13^ To protect the training relationship, Davis and colleagues found that providing feedback about trainee strengths can further the development of the supervisor-learner rapport.^14^

In contrast to time constraints and fear of damaging rapport, the resistant trainee can pose a challenging barrier because it is beyond the supervisor's direct control.^14^ Feedback theory has shown that when feedback is delivered directly, some learners become defensive, and feedback is no longer effective.^13^ To overcome this barrier, research supports starting from a place of trainee self-assessment.^15^^,^^16^  Once trainees share their self-assessment, supervisors are more able to assess a trainee's level of insight. Telio and colleagues state that feedback should be a bidirectional conversation rather than a monologue;^17^ asking resistant trainees how they prefer to receive feedback allows them to be engaged in the process. When corrective feedback must be delivered, Jug and colleagues recommend framing the feedback from a subjective point of view and limiting feedback to 1 or 2 pieces at a time.^18^ Finally, supervisors who are open to receiving feedback from the trainee about their performance will further support normalizing a culture of feedback.

The final feedback barrier to overcome is a supervisor's own discomfort with feedback delivery. If feedback is delivered poorly, Mitchell and colleagues have demonstrated that it can be a negative experience for the trainee.^19^ Feedback research in medical education supports moving away from constructive criticism and towards a focus on effective feedback. Supervisors must be deliberate when selecting a feedback approach to avoid perpetuating ineffective styles they experienced during their training. The best way to overcome the discomfort with feedback delivery is to develop a consistent, structured approach. Learning theory has shown that models based on a trainee's goals can be highly effective.^20^^,^^21^ Offering a trainee education on how to set effective goals using the SMART goals model is an evidence-based place to start.^22^ This framework is designed to make sure goals are: Specific, Measurable, Assignable, Realistic and Time-related. Once SMART goals are developed, there are evidence-based feedback models that can be implemented. The "R2C2" model includes the following 4 phases: rapport building, exploring reactions to feedback, exploring feedback content and coaching for change.^23^ The strength of this model lies in the emphasis on building relationships and approaching feedback as coaching. The SET-GO model uses a trainee driven, descriptive approach centered on outcomes. It stands for: describing what you saw as the supervisor, what else did you see (expounding), what did the learner think, what goal would we like to achieve, and any offers of how we should get there.^24^ The strengths of SET-GO include providing feedback about behavior rather than character and allowing the trainee to participate actively in the feedback process. We have developed a structured feedback delivery model that combines self-determined learner goals with supervisor guided objectives.^25^ The strength of this model is supervisors are able to encourage the selection of objectives that align with both the learner's goals and supervisor perceived deficits.

## Conclusions

Medical education research has demonstrated that feedback is a crucial part of the learning process. Despite this, we know that supervisors experience ongoing challenges to feedback delivery. We propose that overcoming barriers to feedback can only begin when supervisors engage in a meaningful self-inventory of their experience with feedback. Understanding the unique obstacles to feedback delivery each supervisor experiences will allow them to utilize the relevant evidence-based solutions summarized in this article. In addition to implementing these proposed solutions on an individual level, we believe that faculty development modules can be designed and disseminated based on this research.

### Conflict of Interest

The authors declare that they have no conflict of interest.
